# Electronic Nose Analysis of Exhaled Breath Volatile Organic Compound Profiles during Normoxia, Hypoxia, and Hyperoxia

**DOI:** 10.3390/molecules29184358

**Published:** 2024-09-13

**Authors:** Pasquale Tondo, Giulia Scioscia, Marcin Di Marco, Vitaliano Nicola Quaranta, Terence Campanino, Giuseppe Palmieri, Andrea Portacci, Andrea Santamato, Donato Lacedonia, Giovanna Elisiana Carpagnano, Silvano Dragonieri

**Affiliations:** 1 Respiratory Diseases, Department of Medical and Surgical Sciences, University of Foggia, 71122 Foggia, Italy; pasquale.tondo@unifg.it (P.T.); giulia.scioscia@unifg.it (G.S.); campanino.terence@gmail.com (T.C.); giuseppe_palmieri.552807@unifg.it (G.P.); donato.lacedonia@unifg.it (D.L.); 2 Respiratory Diseases, Department DiBrain, University of Bari, 70124 Bari, Italy; marcindimarco@gmail.com (M.D.M.); vitalianonicola.40@gmail.com (V.N.Q.); a.portacci01@gmail.com (A.P.); elisiana.carpagnano@uniba.it (G.E.C.); 3 Unit Physical Medicine and Rehabilitation Section, Department of Medical and Surgical Sciences, University of Foggia, 71122 Foggia, Italy; andrea.santamato@unifg.it

**Keywords:** volatile organic compounds, exhaled breath, electronic nose, hypoxia, hyperoxia

## Abstract

This study investigates volatile organic compound (VOC) profiles in the exhaled breath of normal subjects under different oxygenation conditions—normoxia (FiO2 21%), hypoxia (FiO2 11%), and hyperoxia (FiO2 35%)—using an electronic nose (e-nose). We aim to identify significant differences in VOC profiles among the three conditions utilizing principal component analysis (PCA) and canonical discriminant analysis (CDA). Our results indicate distinct VOC patterns corresponding to each oxygenation state, demonstrating the potential of e-nose technology in detecting physiological changes in breath composition (cross-validated accuracy values: FiO2 21% vs. FiO2 11% = 63%, FiO2 11% vs. FiO2 35% = 65%, FiO2 21% vs. FiO2 35% = 71%, and *p* < 0.05 for all). This research underscores the viability of breathomics in the non-invasive monitoring and diagnostics of various respiratory and systemic conditions.

## 1. Introduction

The analysis of exhaled breath has emerged as a non-invasive diagnostic tool with the potential to provide insights into a wide range of physiological and pathological conditions. Exhaled breath contains a complex mixture of volatile organic compounds (VOCs) that are byproducts of metabolic processes occurring within the body. These VOCs can serve as biomarkers for various diseases and conditions, making breath analysis a promising field for the early diagnosis and monitoring of health status [1,2].

The electronic nose (e-nose) is an innovative technology designed to detect and discriminate among complex odors and VOC profiles. Unlike traditional analytical techniques, such as gas chromatography–mass spectrometry (GC-MS), which are time-consuming and require extensive sample preparation, e-noses offer rapid, real-time analysis with minimal preparation [3]. E-noses comprise an array of sensors that respond to the chemical composition of exhaled breath, producing unique electronic signals or “smell prints” that can be analyzed and classified using advanced statistical and machine learning techniques [3].

Oxygenation states, specifically normoxia, hypoxia, and hyperoxia, reflect the varying levels of oxygen available in the blood. Normoxia refers to a state in which the levels of oxygen in a given environment (e.g., tissue, blood, or atmosphere) are within the normal range for a particular organism or condition. For humans, this typically means oxygen partial pressure (PaO2) in arterial blood of approximately 75–100 mmHg at sea level, typically corresponding to a fraction of inhaled oxygen (FiO2) of 21%, which is equivalent to the oxygen concentration in ambient air. Hypoxia is a condition where there is a deficiency of oxygen in the tissues or a low level of oxygen in the environment compared to normal levels, which can result from conditions such as chronic obstructive pulmonary disease (COPD), sleep apnea, and acute respiratory distress syndrome (ARDS). Hyperoxia refers to an excess of oxygen in the tissues or an environment where the oxygen concentration is higher than normal. In the context of arterial blood, hyperoxia typically refers to an oxygen partial pressure (PaO2) significantly higher than the normal range, often due to supplemental oxygen or other interventions [4]. While oxygen is essential for life, hyperoxia can be harmful and lead to oxidative stress, cellular damage, and various complications, particularly in certain medical contexts or due to prolonged exposure [5].

Although pulse oximetry offers a rapid and non-invasive method for assessing oxygenation levels, the use of an e-nose provides additional diagnostic value by capturing subtle variations in VOC profiles. These variations can reflect underlying metabolic processes, offering a deeper understanding of the physiological changes associated with different oxygenation states.

Understanding how different oxygenation states affect VOC profiles in exhaled breath is crucial for developing diagnostic and monitoring tools. Changes in oxygen levels can influence metabolic pathways and the production of VOCs, potentially providing biomarkers for different physiological and pathological conditions [4].

Previous studies have demonstrated the utility of e-noses in detecting and differentiating between various diseases through breath analysis [6]. For instance, e-noses have been used to distinguish between lung cancer patients and healthy controls as well as between individuals with and without head and neck cancer [7,8] to identify bacterial infections [9] and to monitor metabolic disorders, such as diabetes [10]. However, there is limited research focusing on how different oxygenation states specifically affect VOC profiles in exhaled breath.

The primary objective of this study is to investigate VOC profiles in the exhaled breath of normal subjects under three different oxygenation states: normoxia (FiO2 21%), hypoxia (FiO2 11%), and hyperoxia (FiO2 35%). Using an e-nose, we intend to identify possible differences in VOC patterns among these conditions. Notably, the aim of this investigation is not to compare the use of e-nose technology with pulse oximetry, which is a well-established tool for measuring oxygenation levels in the blood. Instead, we aim to assess whether different oxygen concentrations may influence the spectrum of exhaled VOCs. This is a key methodological question, as understanding how oxygenation affects VOC profiles is critical for future studies involving the e-nose.

## 2. Results

Baseline characteristics showed a predominant number of males, normal lung function and Body Mass Index (Table 1). 

The principal component analysis identified four principal components (PCs). PC1 accounted for most of the variance, explaining 97.619% of the total variance. PC4 was the only component found to be significant in the ANOVA test and was, thus, included alongside PC1 in the discriminant analysis.

The groups were defined as follows: Group 0 (FiO2 21%), Group 1 (FiO2 11%), and Group 2 (FiO2 35%). When comparing these groups across the four principal components, no significant differences were observed in PC1, PC2, and PC3 among the groups, as their *p*-values were 0.991, 0.513, and 0.429, respectively. However, a significant difference was found in PC4, with a *p*-value of 0.000. Specifically, Group 0 differed significantly from both Group 1 and Group 2 in PC4, and Group 1 differed significantly from Group 2 in PC4.

**Figure 1:**
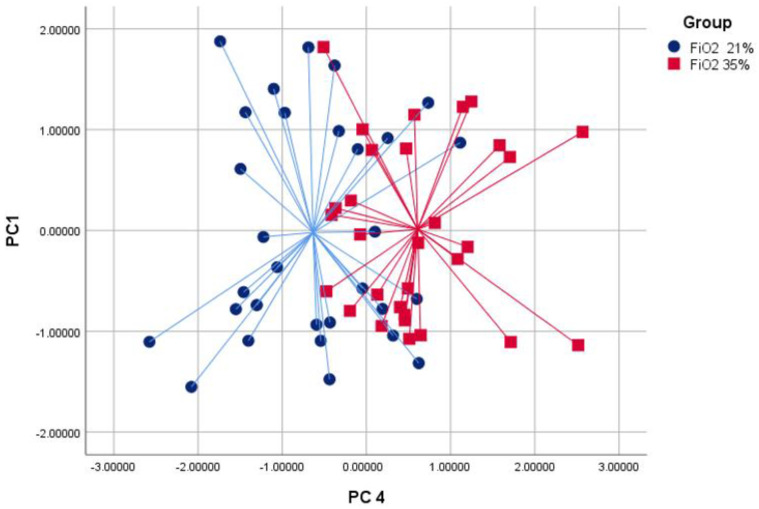
Discriminant analysis results comparing groups with FiO2 levels of 21% (bullets) and 35% (squares) using principal components 1 and 4. The cross-validated value for distinguishing between these two groups is 71% (*p* < 0.05).

**Figure 2:**
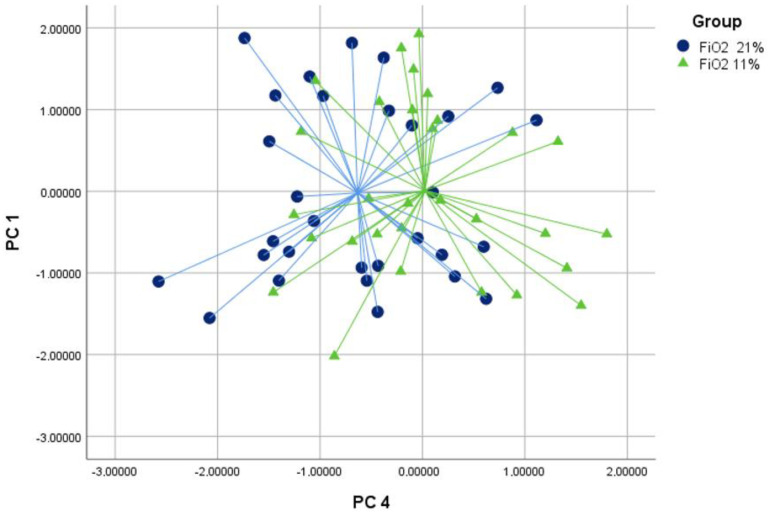
Discriminant analysis comparing groups with FiO2 levels of 21% (bullets) and 11% (triangles) based on principal components 1 and 4. The analysis achieved a cross-validated value of 63% (*p* < 0.05).

**Figure 3:**
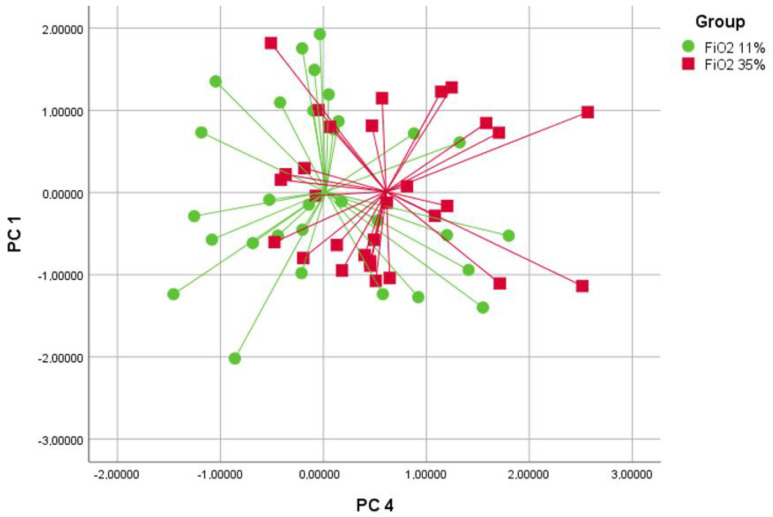
Discriminant analysis comparing groups with FiO2 levels of 11% (bullets) and 35% (squares) using principal components 1 and 4. The cross-validated value for this comparison is 65% (*p* < 0.05).

The discriminant analysis used PC1 and PC4 to differentiate between the groups. The cross-validated accuracy values for discriminating between the groups were 71% for FiO2 21% versus FiO2 35% (*p* < 0.05, Figure 1), 63% for FiO2 21% versus FiO2 11% (*p* < 0.05, Figure 2) and 65% for FiO2 11% versus FiO2 35% (*p* < 0.05, Figure 3).

## 3. Discussion

The present study aimed to investigate the VOC profiles in the exhaled breath of healthy individuals under different oxygenation states—normoxia (FiO2 21%), hypoxia (FiO2 11%), and hyperoxia (FiO2 35%)—using an electronic nose (e-nose). Our findings demonstrate significant differences in VOC patterns among the three conditions, highlighting the potential of e-nose technology to detect physiological changes related to varying oxygen levels. This research holds significant potential for advancing the field of breathomics and non-invasive diagnostics. By elucidating the effects of different oxygenation states on exhaled VOC profiles, we could improve the accuracy and reliability of e-nose-based diagnostic tools. This study also contributes to the broader understanding of metabolic changes associated with varying oxygen levels, which can have implications for the management of respiratory and systemic diseases.

Our results show distinct VOC profiles corresponding to each oxygenation state. principal component analysis (PCA) and canonical discriminant analysis (CDA) revealed significant variations, particularly for principal component 4 (PC4), which exhibited notable differences across the groups. The ANOVA test confirmed these differences, and subsequent pairwise comparisons identified specific group differences. The CDA further supported the classification accuracy between the different oxygenation states, with cross-validated values indicating the ability to differentiate between normoxia, hypoxia, and hyperoxia with reasonable accuracy.

The observed variations in VOC profiles can be attributed to the metabolic changes induced by different oxygen levels. The e-nose’s ability to detect complex VOC patterns provides a non-invasive means of monitoring metabolic changes, potentially offering early indicators of hypoxia or hyperoxia that are not detectable through traditional pulse oximetry. Hypoxia and hyperoxia are known to influence cellular metabolism and oxidative stress, which, in turn, affect the production and release of VOCs [4,5]. For instance, hypoxia can lead to increased anaerobic metabolism and the production of metabolites, such as lactic acid, which may alter the VOC composition in exhaled breath [4]. Hyperoxia, on the other hand, can enhance oxidative metabolism and the generation of reactive oxygen species (ROS), potentially leading to distinct VOC patterns [4,5]. The statistically significant changes in VOC profiles, although subtle, may serve as early indicators of physiological shifts that precede clinical symptoms. These findings could be pivotal in developing non-invasive diagnostic tools for the early detection of hypoxia and hyperoxia.

To the best of our knowledge, this is the first study specifically addressing the differences in exhaled VOC profiles under normoxia, hypoxia, and hyperoxia using an electronic nose in healthy subjects.

Several previous studies provide additional context to our findings. Lacey et al. [11] investigated the use of an e-nose to detect hypoxia-induced changes in VOC profiles at high altitudes and demonstrated that e-nose technology could identify individuals at risk of acute mountain sickness (AMS) by analyzing breath samples [11]. This study aligns with our findings, indicating that hypoxic conditions significantly alter VOC profiles, which can be detected by e-nose technology. Relatedly, Mazzatenta et al. [12] investigated VOC changes in subjects experiencing hypoxia triggered by pathological conditions rather than by breathing in a controlled hypoxic mixture, as in our study. While their study demonstrated significant changes in VOC profiles under hypoxic conditions, it is important to note that other underlying pathological processes may have contributed to these changes [12].

Another relevant study by Harshman et al. [13] identified hypoxia biomarkers from exhaled breath under normobaric conditions using gas chromatography–mass spectrometry (GC-MS). They discovered significant changes in specific VOCs, including pentanal, 4-butyrolactone, 2-pentanone, 2-hexanone, 2-cyclopenten-1-one, 3-methylheptane, and 2-heptanone, in response to hypoxic conditions [13]. This study supports our findings by demonstrating the changes in VOC profiles due to hypoxia and highlights the potential of VOC analysis in the non-invasive monitoring of hypoxic conditions.

Concerning hyperoxia, Cronin et al. identified GC-MS 18 VOC biomarkers that precede pulmonary oxygen toxicity (PO2T) in a swine model exposed to prolonged hyperoxia, with six VOCs being particularly predictive of PO2T [14]. The predictive breath test developed from these findings showed potential for the early detection of PO2T [14]. Moreover, de Jong et al. focused on VOCs identified through GC-MS analysis and identified a collection of VOCs, primarily methyl alkanes, associated with hyperbaric hyperoxia [15]. Although no single marker was universally present across all studies, the identified VOCs provided a robust set of potential biomarkers for PO2T, confirming that a combination of VOCs, rather than a single marker, is likely necessary to assess hyperbaric oxidative stress [15]. Finally, van Ooij et al. investigated the effects of hyperbaric oxygen exposure on exhaled VOCs in male divers using GC-MS and identified significant changes in VOC profiles after oxygen dives, including a particular increase in methyl alkanes, suggesting a distinct VOC breath print associated with hyperbaric oxygen exposure [5].

The ability to non-invasively monitor changes in VOC profiles related to oxygenation states has significant clinical implications. For patients with respiratory conditions, such as COPD or ARDS, the regular monitoring of exhaled breath could provide valuable insights into their oxygenation status and metabolic health. The use of e-nose technology could facilitate the early detection of hypoxia or hyperoxia, allowing for timely interventions and better management of these conditions.

Moreover, the differentiation of VOC profiles under varying oxygen levels could enhance the diagnostic capabilities of e-noses. By incorporating specific VOC markers associated with different oxygenation states, e-noses could improve the accuracy and reliability of breath-based diagnostics. This advancement could extend to other clinical scenarios, such as perioperative monitoring and critical care, where maintaining optimal oxygen levels is crucial.

Despite the promising results, our study has several limitations. The sample size was relatively small, which may limit the generalizability of the findings. Future studies with larger cohorts are necessary to validate the observed differences in VOC profiles. Additionally, while the e-nose provides rapid and real-time analysis, it lacks the specificity of traditional analytical techniques like GC-MS. Further research is needed to identify and quantify the specific VOCs contributing to the observed patterns.

One potential limitation of this study lies in the sequence of administering oxygenation conditions, specifically the choice to expose participants to hypoxia first, followed by hyperoxia. This decision was made primarily for patient safety, as inducing hypoxia poses a greater immediate risk and requires careful monitoring and control. However, we acknowledge that this sequential design could introduce a bias, as the metabolic changes induced by hypoxia might influence the subsequent VOC measurements during hyperoxia. A crossover design for future studies, in which half of the subjects receive hypoxic conditions first, and the other half begin with hyperoxic conditions, could mitigate such potential bias by balancing the order of exposure.

Furthermore, this study focused solely on healthy individuals. Investigating VOC profiles in patients with respiratory or systemic diseases under different oxygenation conditions could provide more comprehensive insights and enhance the clinical applicability of e-nose technology. The Cyranose 320 e-nose, equipped with an array of 32 polymer sensors, is designed to detect a broad range of VOCs. However, its sensitivity to specific oxygen-induced changes in VOC profiles remains an area for further exploration. Our findings suggest that the device can indeed detect these changes, albeit with certain limitations that warrant additional investigation. Finally, a key limitation to this study is the arbitrary choice of FiO2 values for hypoxia and hyperoxia. This choice may influence the results, and we cannot exclude the possibility that different FiO2 values might yield different outcomes. Additional studies with a more systematic approach to selecting FiO2 values are necessary to validate these findings. Similarly, the 5 min duration for each oxygenation cycle was selected to balance participant safety with the need to induce detectable VOC changes. While this duration was sufficient for our study, future research should investigate the effects of prolonged exposure to better understand the temporal dynamics of VOC production under different oxygenation states.

Future research should aim to expand the sample size and include diverse populations to validate and generalize the findings. Exploring the specific VOCs responsible for the observed differences, using complementary techniques such as GC-MS, could provide deeper insights into the metabolic changes associated with varying oxygen levels.

Additionally, integrating machine learning algorithms with e-nose data could enhance the classification accuracy and predictive capabilities of the device. Developing standardized protocols for breath sample collection and analysis could also improve the reproducibility and reliability of e-nose-based diagnostics.

## 4. Materials and Methods

### 4.1. Participants

A total of 30 healthy volunteers (20 males and 10 females) participated in this study. All participants were free from any history of chest symptoms and systemic diseases, and none were taking any medications. The age range was 22 to 45 years, and all subjects exhibited normal lung function. Individuals with a history of respiratory tract infections in the four weeks preceding this study were excluded. Exhaled breath samples were collected from all subjects under three different oxygenation conditions: normoxia (FiO2 21%), hypoxia (FiO2 11%), and hyperoxia (FiO2 35%). The participants were recruited from hospital staff, and participation was voluntary. The study was approved by the ethics committee of Bari Policlinico (protocol number 46403/15), and all participants provided informed consent before participating.

### 4.2. Study Design

A longitudinal study design was employed. Participants attended two separate sessions to complete all measurements. During the first visit, subjects were screened according to inclusion/exclusion criteria, and those who qualified underwent a flow-volume spirometry test (MasterscreenPneumo, Jaeger, Würzburg, Germany). Normal lung function was confirmed through spirometry, using Global Lung Function Initiative (GLI) reference values to ensure consistency across assessments. During the second visit, exhaled breath samples were collected after normoxia and during induced hypoxia and hyperoxia states. The breath samples were immediately analyzed using the e-nose after each phase. Participants were allowed to drink only still water on the day of testing and could not perform physical exercise nor brushing teeth with toothpaste. Exhaled breath was collected while subjects wore a nose clip throughout the procedure. Initially, there was a 1 min wash-in period with a 3-way non-rebreathing valve coupled to an inspiratory VOC filter (A2; North Safety, Middelburg, The Netherlands) to minimize environmental VOC contamination and to ensure baseline stabilization. Measurements were consistently performed at the same time of day to control potential diurnal variations in VOC levels. Subjects then exhaled a vital capacity into a Tedlar bag, which was promptly sampled by the e-nose.

### 4.3. Hypoxia and Hyperoxia Induction

Hypoxia and hyperoxia cycles were induced using an intermittent hypoxic-hyperoxic training (IHHT) device called CellOxy^TM^ (Rostock, Germany). This device allows for the precise control of the oxygen content in inhaled air, facilitating cycles of hypoxia (from 9% to 16% FiO2) and hyperoxia (from 24% to 36% FiO2). Each cycle of hypoxia and hyperoxia lasted 5 min, followed by e-nose sampling, with participants remaining connected to the IHHT. The CellOxy device monitors adjusts the oxygen concentration in real time, ensuring the consistent and accurate induction of the desired oxygenation states. Importantly, no participants had to interrupt the procedure due to side effects, indicating that the protocol was well-tolerated. Moreover, no physical effects were observed after the procedures by all individuals.

### 4.4. Electronic Nose

**Figure 4:**
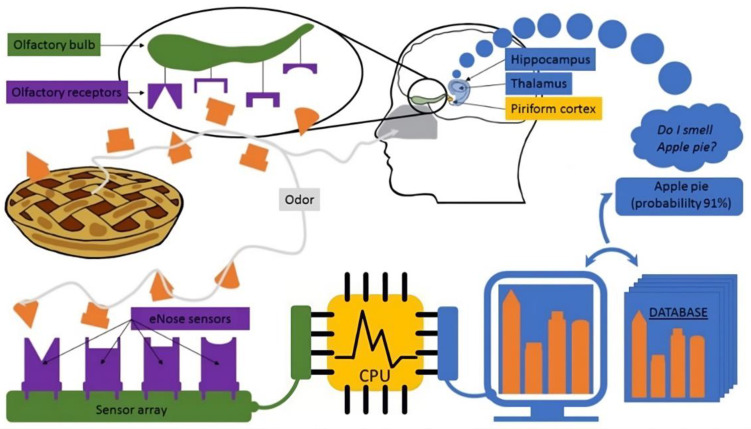
Comparison between the biological olfactory system and an artificial electronic nose (e-nose) system for detecting odors.

We used a Cyranose 320 electronic nose (Sensigent, Irwindale, CA, USA) for this study. This device features 32 organic polymer sensors arranged in a nano-composite array. When exposed to VOCs, these polymers swelled, altering their electrical resistance. The device recorded the raw data (expressed as dR = (R − Ro)/Ro, where R is the response to the sample gas, and Ro is the baseline reading, with ambient room air as the reference gas) from each of the 32 sensors, which was stored in an onboard database. These data create a unique “breathprint” representing the VOC spectrum, which can be analyzed using pattern recognition algorithms (Figure 4).

To ensure accurate measurements, we adhered to the recommended operating parameters from the instruction manual. The baseline purge lasted 30 s at a low pump speed. The sampling time was 60 s at a medium pump speed, followed by a 200 s purge at a high pump speed. Each sample run lasted a total of 300 s, and the device operated at 42 °C. Between samples, a 5 min post-run purge was conducted. Additionally, before the first sample of the day, the sensors were stabilized by exposure to the room air for 5 min, followed by a “blank measurement.” The relative humidity during sample analysis was approximately 55%.

### 4.5. Statistical Analysis

Statistical analyses were performed using SPSS for Windows 26.0 (SPSS, Chicago, IL, USA). The Kolmogorov–Smirnov and Shapiro–Wilk tests were used to assess data distribution. Categorical variables were analyzed using the chi-square test or Fisher’s exact test as appropriate and reported as n (%). Continuous variables were analyzed using ANOVA and Student’s *t*-test for independent samples, with normally distributed continuous parameters reported as the mean (standard deviation [SD]).

Principal component analysis was used to reduce data dimensions. All four principal components (PCs) were compared at different times using the ANOVA test. Here, the ANOVA test indicated significance, and individual groups were compared two-by-two using Student’s *t*-test for independent samples. Canonical discriminant analysis (CDA) was employed to categorize VOC patterns based on the principal components. The leave-one-out validation method was used to calculate the cross-validated accuracy percentage (CVA%), which estimated the predictive model’s practical accuracy. The sample size was determined to limit the standard error to 10%, assuming an 80% accuracy rate, and the current sample size per subgroup was adequate. A *p*-value of <0.05 was considered statistically significant.

## 5. Conclusions

In conclusion, our study demonstrates significant differences in exhaled breath VOC profiles under normoxia, hypoxia, and hyperoxia using an e-nose. These findings highlight the potential of e-nose technology to non-invasively detect physiological changes related to oxygen levels. The clinical implications of this research are vast, offering promising avenues for the early diagnosis and monitoring of respiratory and systemic conditions. Further research is warranted to validate these findings and explore their applicability in clinical settings.

## Figures and Tables

**Table 1:** Baseline characteristics of study population. Values are intended as mean ± standard deviation.

Parameter	Value
Number	30
Age (years)	29.9 ± 7.8
Males (n)	20
FEV1 (%pred)	99.2 ± 0.3
FVC (%pred)	103.1 ± 0.4
BMI (kg/m^2^)	24.8 ± 3.9

## Data Availability

The dataset is available upon appropriate request to the corresponding author.
